# Population Genetics of the Filarial Worm *Wuchereria bancrofti* in a Post-treatment Region of Papua New Guinea: Insights into Diversity and Life History

**DOI:** 10.1371/journal.pntd.0002308

**Published:** 2013-07-11

**Authors:** Scott T. Small, Akshaya Ramesh, Krufinta Bun, Lisa Reimer, Edward Thomsen, Manasseh Baea, Moses J. Bockarie, Peter Siba, James W. Kazura, Daniel J. Tisch, Peter A. Zimmerman

**Affiliations:** 1 Center for Global Health and Diseases, Case Western Reserve University, Cleveland, Ohio, United States of America; 2 Papua New Guinea Institute of Medical Research, Goroka, Papua New Guinea; 3 Centre for Neglected Tropical Diseases, Liverpool School of Tropical Medicine, Liverpool, United Kingdom; University of Melbourne, Australia

## Abstract

**Background:**

*Wuchereria bancrofti* (Wb) is the primary causative agent of lymphatic filariasis (LF). Our studies of LF in Papua New Guinea (PNG) have shown that it is possible to reduce the prevalence of Wb in humans and mosquitoes through mass drug administration (MDA; diethylcarbamazine with/without ivermectin). While MDAs in the Dreikikir region through 1998 significantly reduced prevalence of Wb infection, parasites continue to be transmitted in the area.

**Methods:**

We sequenced the Wb mitochondrial Cytochrome Oxidase 1 (CO1) gene from 16 people infected with Wb. Patients were selected from 7 villages encompassing both high and moderate annual transmission potentials (ATP). We collected genetic data with the objectives to (i) document contemporary levels of genetic diversity and (ii) distinguish between populations of parasites and hosts across the study area.

**Principle Findings:**

We discovered 109 unique haplotypes currently segregating in the Wb parasite population, with one common haplotype present in 15 out of 16 infections. We found that parasite diversity was similar among people residing within the same village and clustered within transmission zones. For example, in the high transmission area, diversity tended to be more similar between neighboring villages, while in the moderate transmission area, diversity tended to be less similar.

**Conclusions:**

In the Dreikikir region of PNG there are currently high levels of genetic diversity in populations of Wb. High levels of genetic diversity may complicate future MDAs in this region and the presence of dominant haplotypes will require adjustments to current elimination strategies.

## Author Summary

The Global Program to Eliminate Lymphatic Filariasis (LF), initiated by the World Health Organization (WHO), aims to eliminate LF from endemic regions, where 1.34 billion people live at risk of this disease. The causative agent responsible for 90% of LF is the nematode parasite species *Wuchereria bancrofti* (Wb). The primary approach to LF elimination has been through mass drug administration (MDA), which serves to interrupt transmission by killing the microfilaria required to continue the parasite life cycle through mosquito transmission. Despite success of MDA, evidence indicates that transmission can rebound if drug administration is discontinued. In the void of well-characterized genetic markers, it is difficult to understand how a Wb population will be impacted by or recover from MDA. Here we use recently described mitochondrial DNA polymorphisms to evaluate the diversity of a Wb population that has been previously exposed to MDA in Papua New Guinea. Our data analyses reveal significant genetic diversity and evidence that MDA has not significantly reduced the genetic complexity of the Wb population. This study describes a population genetic approach for assessing the impact of MDA and other transmission control strategies.

## Introduction

Lymphatic-dwelling nematodes that cause damage to the lymphatic system (lymphatic filariasis—LF) contribute to significant permanent and long-term disability in the world, second only to mental illness [1]. Acute and chronic morbidity resulting from LF has affected 120 million people living in 81 countries with 1.34 billion people at risk of developing infection [2]. In 2000, the World Health Organization (WHO) initiated the Global Program to Eliminate Lymphatic Filariasis (GPELF) with the goal to eradicate LF by 2020. The primary approach to LF elimination has been through mass drug administration (MDA), which serves to interrupt transmission by treating the transmission stage of the infection (microfilaria; MF). In the first 10 years of GPELF activity, more than 3.4 billion treatments were administered to nearly 897 million people in 52 of the 81 endemic countries [2]. Complete MDA programs have now been developed in more than 50 of the LF-endemic countries with 13 of these reaching the goals set forth by the GPELF in all or part of the country [2].

As some countries approach completion of MDA programs, priorities have changed to focus on monitoring elimination success through development of post-MDA surveillance tools [3], [4]. Current surveillance tools include both DNA and antigen-based diagnostics, but these methodologies vary in both specificity and sensitivity [5]–[8]. The most recent generation of surveillance tools has shown improvements in sensitivity and in specificity by differentiating between the three species that cause LF: *W. bancrofti* (Wb), *Brugia malayi*, and *Brugia timori* [9]–[11]. To date, the GPELF has had widespread success using diagnostic assays limited solely to the detection of Wb, however, with much of the parasite's life history still unknown, current strategies may prove insufficient to achieve elimination [12]–[17]. For example, in Haiti Wb prevalence rebounded when annual MDA was missed in one year of a multi-year MDA program [18]. A similar phenomena has also been observed in Papua New Guinea (PNG), where prevalence of LF among 6.3 million inhabitants ranges from 10 to over 90% [19]. In the Dreikikir District of East Sepik Province our five year randomized drug trial (1993–1998) documented a decrease in MF prevalence and transmission by 77–97% [20], [21] but did not halt parasite transmission at sites revisited in 2008.

Through genotyping populations of Wb, we can acquire additional information that is likely to contribute to elimination success. This allows us to move beyond mere detection and track individual Wb strain prevalence through time. Until recently, the availability of genetic markers for differentiating between strains of Wb has been limited, so it has not been possible to evaluate changes in parasite populations in the context of LF elimination programs. Through our recent sequencing of the Wb mitochondrial genome (mtGenome) we have identified numerous genetic polymorphisms that can be used to evaluate population structure and to characterize infection complexity [22]. Here we demonstrate the utility of population genetic studies on populations of Wb in regard to the impact of MDA in the well-studied populations of Dreikikir, PNG through analysis of mtDNA *cytochrome oxidase I* gene polymorphisms. Our results provide the first description of genetic diversity in a Wb parasite population by: *i)* constructing a haplotype network of the infecting strains, *ii)* quantifying the diversity of Wb at both the infrapopulation and host village level, and *iii)* determining if the population is genetically structured. Finally, we demonstrate how genetic diversity data can be used to aid in LF elimination efforts by reconstructing the parasite population history in light of the recent MDA efforts in this region.

## Materials and Methods

### Study site

**Figure 1:**
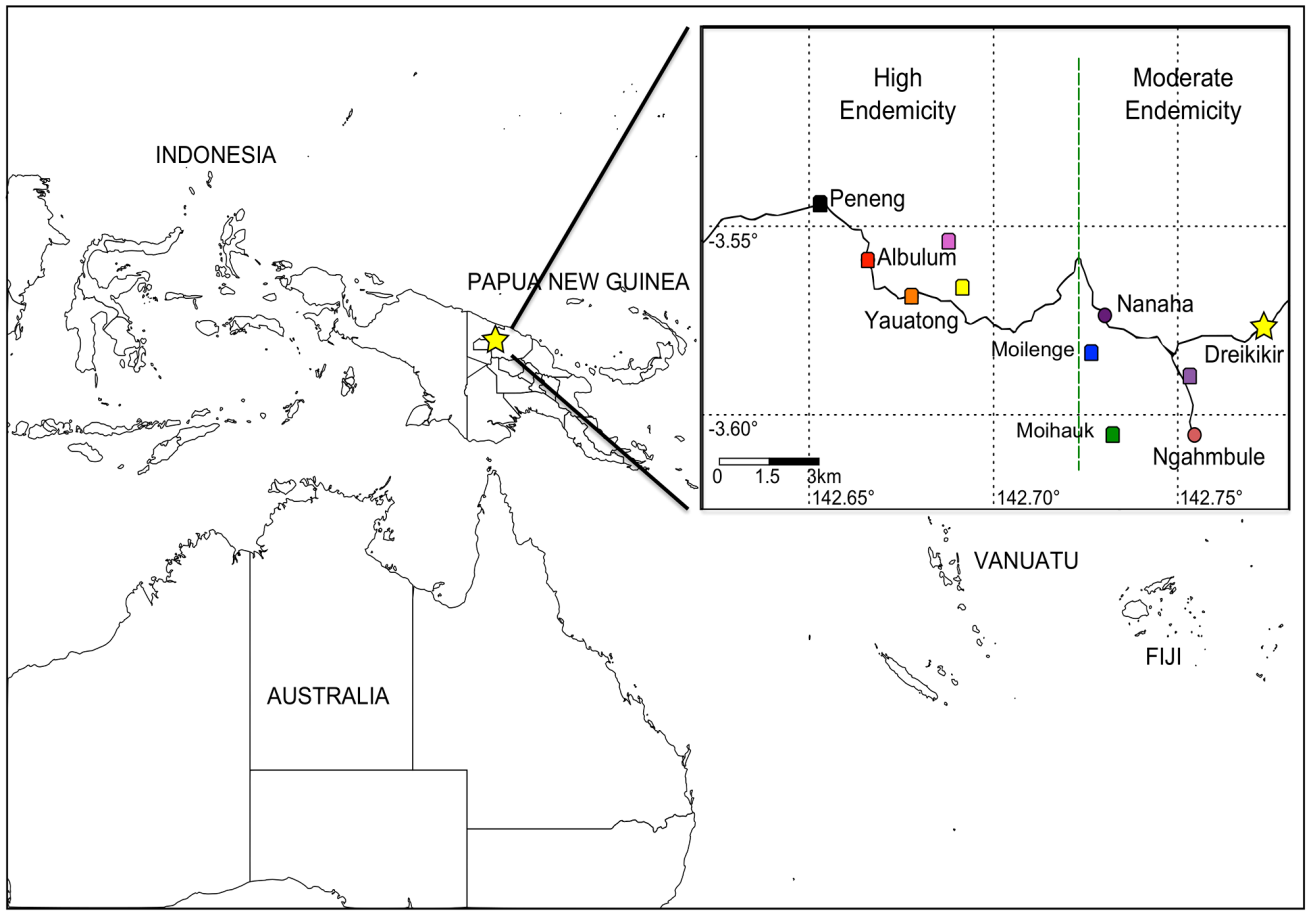
Map of the Dreikikir study site. The map identifies the location of all seven villages included in this study plus two villages used for vector surveys (Nanaha and Nagambuhle) and the location of Dreikikir district station. Albulum includes Albulum 1 (red) and Albulum 2 (pink); Yauatong includes Yauatong 1 (orange) and Yauatong 2 (yellow). The dashed line (green) shows a previously identified division between high and moderate zones of LF transmission.

In 1993, 14 communities in Dreikikir District (population ∼3,500 people), East Sepik Province of Papua New Guinea, underwent a randomized field trial of MDA [Diethylcarmabazine (DEC) with/without ivermectin] to reduce the prevalence of Wb in human and mosquito infections [20], [21] (Figure 1). Over the course of 5 consecutive treatments, spanning the years 1993–1998, MF prevalence and annual transmission potential decreased by 77–97%. Study sites in PNG were revisited in 2008 and whole blood samples were collected under clinical protocols approved by the institutional review boards at the PNG Institute of Medical Research (PNGIMR) and University Hospitals Case Medical Center. Whole blood samples were screened for Wb positivity by a post-PCR ligase detection reaction-fluorescent microsphere assay (LDR- FMA) [23] and blood smear microscopy. A sub-sample of parasites from whole blood positive for Wb were selected from individuals 10–40 years of age with a minimum parasitemia of 50 microfilaria/ml of blood (MF/ml), and reported to have limited migration among study villages over the 10 year post-MDA period (1998–2008). Based on these criteria, sixteen Wb-infected individuals from seven study villages [Peneng (n = 3), Albulum1 (n = 3), Albulum2 (n = 3), Yautong1 (n = 1), Yautong2 (n = 2), Moihuak (n = 2), Moilenge (n = 2)] were included in the study.

### Ethics statement

Institutional review boards (IRBs) of University Hospitals of Cleveland, the PNG Institute of Medical Research, and the PNG Medical Research Advisory Committee approved the study. All study participants provided informed consent; parents/guardians provided consent on behalf of all child participants. Consent for all study participants was written.

### Genomic DNA extraction and PCR amplification

Genomic DNA (gDNA) was extracted from whole blood using a QIAamp 96 DNA Blood Kit (QIAGEN, Valencia, CA). PCR Primers were designed to amplify 690 base pairs of the cytochrome oxidase 1 gene *(CO1)* [22]. All PCR methods, cloning, and modified sequencing reactions can be found in the supplemental methods (Text S1) as well as in Ramesh *et al.* 2012 [22].

### Sequence data processing

All sequences were edited and assembled using CodonCode Aligner 3.5 (CodonCode Corporation, Dedham MA); hereafter a sequence refers to a sequence collected from a single clone. Primer sequences were deleted and remaining sites with PHRED scores <30 were visually inspected and recorded as ambiguities. Sequences with less than a minimum of 300 high quality bases (PHRED score >30) were removed from further analyses. Edited sequences were then imported into Geneious 5.3 and aligned against the complete Wb mitochondrial genome sequence (GenBank Accession No. JF 557722) to verify that all the sequences were in identical translation frames and contained no stop codons. Afterward, a correction for Taq DNA polymerase errors was applied to aligned sequences within each infrapopulation (Wb population within a single human host) (see Text S1, *Sequence processing*). Corrections included removal of polymorphisms that occurred less than twice in individual alignments (treated as *Taq* DNA polymerase errors) and were modified to match the consensus sequence (see Text S1, *Sequence processing*). After correcting polymerase errors, UCHIME, a chimera detection program, was used to detect PCR-based recombinant molecules [24]. Sequences reported as chimeric were subsequently removed from further analysis. All sequences were deposited in Genbank (www.ncbi.nlm.nih.gov/genbank) as of January 2013 as popset (KC558603–KC559091).

### Genetic diversity

Sequence alignments for each parasite infrapopulation were generated using Geneious 5.3 (Biomatters, Auckland, NZ). Pairwise nucleotide diversity (π) [25] and the population mutation rate (*θ*) [26] were calculated using DnaSP 5.0 [27]. Analysis of molecular variance (AMOVA) was used to partition genetic variance—the average genetic distance between randomly chosen haplotypes or alleles—into hierarchical components [28]. Hierarchical components were defined to be: *i)* among parasite infrapopulations (Φ_ST-H_), *ii)* among parasite infrapopulations within a host village (Φ_ST-HV_), and *iii)* among host villages (Φ_ST-V_). AMOVA was performed using Arlequin 3.5 [29] with significance determined by 16,000 permutations.

A multiple linear regression was used to build a predictive model of the number of Wb strains within a host. Here we define strain as a unique haplotype isolated from a single infrapopulation; each unique CO1 haplotype is taken to represent a maternal strain. Individual host factors were compiled into a data frame and analyzed using a generalized linear model, with strains as the dependent factor, in R statistical software. The best-fit model was determined based on the lowest Akaike's Information Criteria (AIC) value as given by step-wise addition and removal. A test for multi-collinearity was performed to exclude any factors inflating variance. Model fit was also visually examined using the CAR package in R. Further tests for violations to linear model assumptions were performed using the GVLMA package in R [30].

### Genetic structure of infrapopulations

Pairwise measures of genetic differentiations used both a comparison of sequence difference among populations as well as the difference in allele frequency among populations. Pairwise genetic differentiation, Φ_ST-H_ [28], was calculated in the program ARLSUMSTAT [29]. Significance was determined by a Kruskal-Wallace test in R with pairwise significance determined using the R package Kruskal MCMC (Bonferroni's adjusted p-value, α = 0.0004). Pairwise allele differentiation, Jost's D (D_J-H_) [31], was calculated using the code from Pennings 2011 [32]. Significance was determined by a permutation test [32] (Null hypothesis D_J-H_ = 0; corrected p-value, α = 0.0004).

### Genetic structure of host villages

Non-significant values of AMOVA analysis, pairwise Φ_ST-V_, and pairwise statistics were used as a measure of panmixia and permitted the grouping of infrapopulations into host villages [Peneng, Albulum1, Albulum2, Yautong2, Moihuak, Moilenge] (see Text S1, *Grouping Infrapopulations*). The host village dataset was assessed for genetic diversity and genetic differentiation following the methods indicated in the infrapopulations section above. The only deviation in analysis was the Bonferroni's adjusted p-value, where α = 0.002 for both the Φ_ST-V_ and D_J-V_ statistics. Genetic differentiation results for host villages were visualized in multi-dimensional scaling plots using XLSTAT Addinsoft software [33]. Details on power tests for detecting genetic differentiation are provided in the supplemental methods (Text S1, *Power Tests of Genetic Differentiation*).

### Phylogenetics

Haplotype networks were created using the program NETWORK 4.6.1.0 (fluxus-engineering.com) using only unique sequences from each infrapopulation. Pre-processing was performed via a Star Contraction to collapse nodes <2 mutational steps apart. Networks were then constructed using median joining with maximum parsimony [34], [35]. Resulting nodes were color-filled to represent host villages and size adjusted to represent the frequency of each resulting haplotype.

### Reconstructing population history after an MDA

Tajima's D statistic [36] compares the number of segregating sites (*S*) to the average number of pairwise mutations (*π*) [25] in a population sample. Since both estimates are unbiased for the population mutation rate parameter *θ* [26], the difference is expected to be 0. The resulting sign and significance of Tajima's D statistic provides information on population history such as population size changes. A Tajima's D of 0 signifies that the population did not experience a population expansion or contraction. In cases where Tajima's D<0 there is an excess of rare mutations that could result from a recent population expansion or positive selection. Where Tajima's D>0, fewer rare mutations are observed than expected, possibly resulting from a recent population contraction or balancing selection. Tajima's D test statistic was calculated for infrapopulations and host villages (after grouping) using DnaSP 5.0 [27] and 1000 coalescent simulations in DnaSP 5.0 were used to determined significance. All populations were also tested using a haplotype-based test, Strobeck's *S* [37] in DnaSP 5.0.

## Results

We collected a total of 487 sequences from 16 infected individuals residing in 7 study villages. These individuals were selected based on age (<40 years of age), migration history (same location in 1998 and 2008), and parasitemia (greater than 50 MF/ml of blood) to represent each host village location. From the total of 487 sequences generated, 109 were identified as unique haplotypes, thereby representing individual strains. A summary of individuals included in the study is found in Table 1.

**Table 1:** Summary statistics for infected individuals.

Village	Transmission Zone	1998 mf prevalence	2008 mf prevalence	Patient ID	MF/ml	Age group (years)	Number of sequences	Number of Haplotypes^A^	π^B^	TajD^C^
Peneng	High	2.83%	23.71%	T0059PN	79	21–30	37	9	1.543	1.67105
				T0083PN	74	21–30	19	4	1.185	0.86015
				T0097PN	66	21–30	13	2	0.222	NA
Albulum 1	High	4.69%	33.33%	T0150A1	972	21–30	55	29	1.753	0.53053
				T0186A1	295	21–30	17	6	0.948	0.49119
				T0388A1	367	21–30	75	19	1.942	1.06353
Albulum 2	High	7.53%	44.66%	T0142A2	74	11–20	47	12	1.323	1.62329
				T0145A2	208	11–20	24	9	1.667	1.32831
				T0346A2	194	11–20	63	29	2.073	0.80478
Yautong 1	High	8.79%	41.18%	T0363Y1	2964	21–30	23	8	1.595	1.67776
Yautong 2	High	17.24%	51.56%	T0557Y2	517	11–20	18	8	1.849	0.70397
				T0582Y2	1476	21–30	24	12	1.886	0.72805
Moilenge	Moderate	0.00%	12.00%	T0609ML	303	31–40	12	8	1.944	1.34788
				T1602ML	369	21–30	13	10	1.684	1.57314
Moihauk	Moderate	1.35%	10.19%	T1358MO	2361	11–20	33	5	2.044	0.06473
				T1384MO	517	11–20	14	8	1.294	0.8157

A Haplotypes identify unique strains.

B Nucleotide diversity as the average proportion of nucleotide differences between all possible pairs of sequences [26].

C Tajima's D test statistic [29] with significance calculated by 1000 coalescent simulations in DnaSP 5.0.

### *W. bancrofti* strain diversity and network relationships

Overall the level of genetic diversity was highly variable among parasite infrapopulations with mean nucleotide diversity (π) varying as much as 9-fold between infections (e.g. T0097PN: π = 0.222% and T0346A2: π = 2.073%) (Table 1). We also found that the number of unique haplotypes, or strains, within host infrapopulations was positively correlated with nucleotide diversity in the same host but was not significant (Pearson correlation r = 0.50, p = 0.061). Nucleotide diversity within host infrapopulations was negatively correlated with the parasitemia, given as MF/ml, (Pearson correlation r = −0.02, p = 0.70) and positively correlated with the number of sequences (Pearson correlation r = 0.82, p<0.001) (Figures S1 & S2). No further correlations were found between patient factors and nucleotide diversity of infrapopulations.

**Figure 2:**
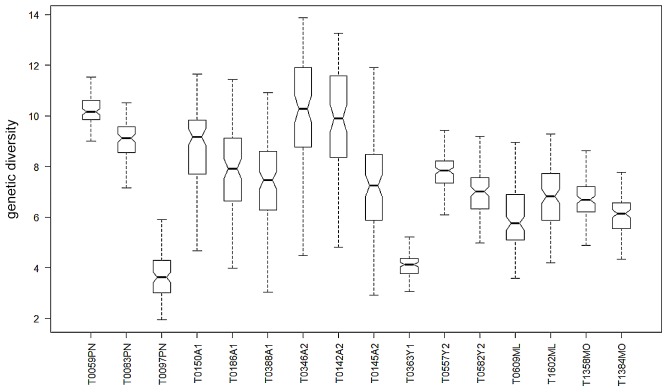
Genetic diversity of infrapopulations. Values of genetic diversity as calculated by the neutral mutation rate (*θ*) [26] for each individuals' infrapopulation across the study area (presented from west to east). A notched box plot represents *θ* values and their distributions with median value shown as darkened horizontal line. The width of the notches is proportional to the interquartile range of the sample and inversely proportional to the square root of the size of the sample. Whiskers represent 1^st^ and 3^rd^ quartiles of the distribution. The corresponding numbers of strains for each infrapopulation are found in Table 1.

Values of diversity given by *θ* were highly variable among the infrapopulations (Figure 2). There were no clear patterns among infrapopulations within host village and in some cases low and high diversity infrapopulations were found in the same villages. For example, in Peneng where the infections analyzed were all characterized by similar levels of microfilaremia (range 66 to 79 MF/mL, Table 1), individuals T0059PN and T0083PN both had highly diverse infections, whereas individual T0097PN harbored one of the least diverse infections. Though not significant, there was a trend for higher *θ* values to the west and lower values to the east of Yautong1.

Consistent with the high levels of diversity observed within infections, the analysis of molecular variance (AMOVA) attributed 90.31% of the genetic variance in the overall parasite population to be within infrapopulations (Φ_ST-H_). The remaining variance was divided among infrapopulations within a host village (Φ_ST-HV_, 2.71%) and among host villages (Φ_ST-V_, 6.98%). Fixation indices were only significant in the case of Φ_ST-H_ (p = 0.019), indicating a significant difference among the ungrouped infrapopulations.

**Figure 3:**
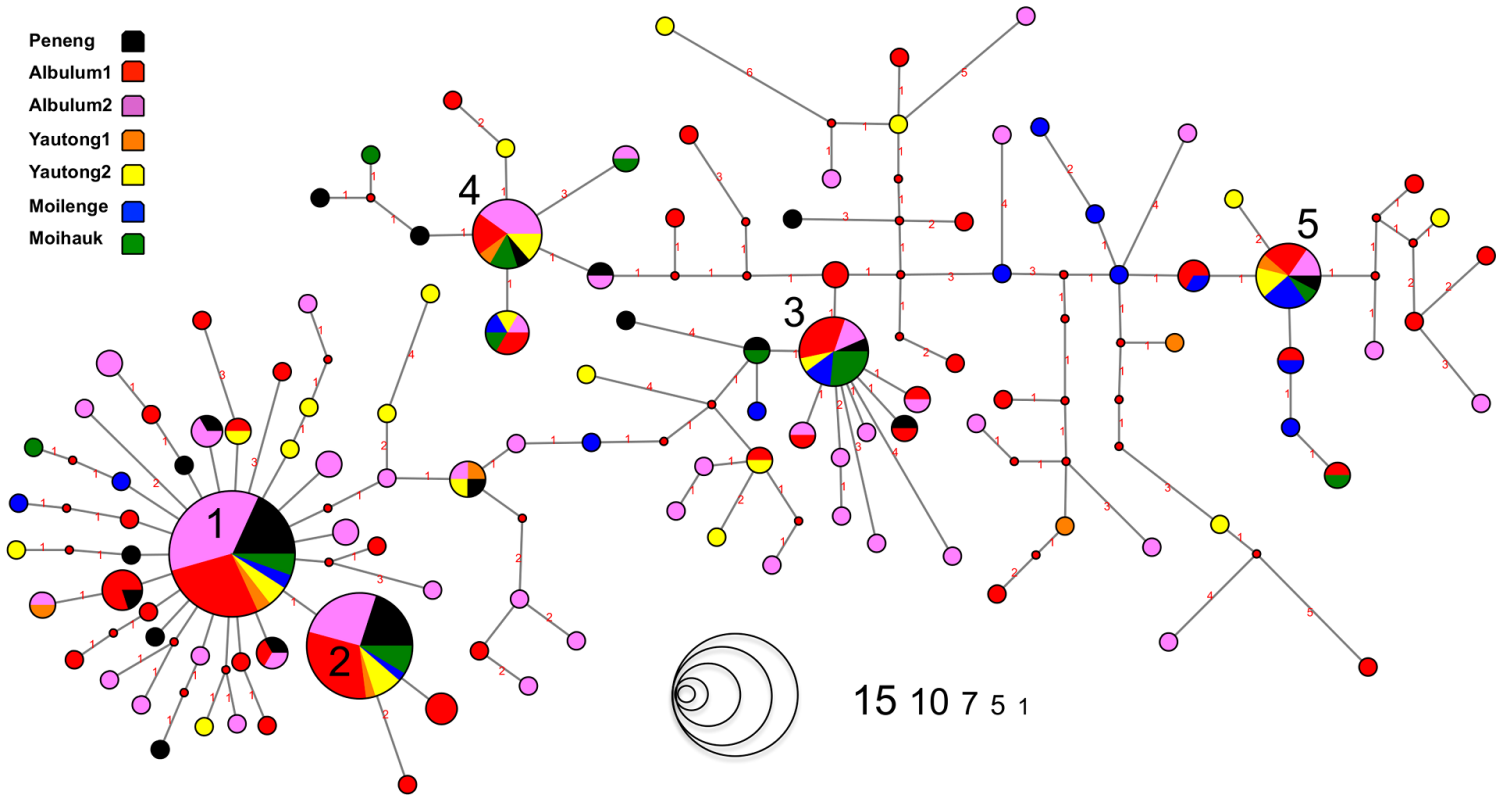
Network analysis of Wb strains. Maximum parsimony network where the nodes are color-coded by host village; color designation corresponds to map and legend in Figure 1. In the haplotype network mutational steps are represented as red numbers placed on the connecting lines. From the total of 487 sequences generated, 109 were identified as unique haplotypes, thereby representing individual strains. Frequencies of each strain, corresponding to the number times that strain was observed in the unique infrapopulation sample of 178 sequences, are represented by circles of known size in lower legend. The five common strains are numbered with the highest frequency strain designated ‘1’ and the lowest frequency being designated ‘5’.

The network analysis (Figure 3) illustrates relationships within the Wb parasite population. In contrast to summary statistics in Table 1, the network adds information on the frequency of each unique haplotype (deemed strain) along with the relatedness (number of mutational steps) separating each strain. There were 5 strains (frequencies >10%) making up 27% of the overall Wb population analyzed, with strains 1 and 2 being the most frequent. Taken together, strains 1 and 2 made up a total of 15% of the Wb population analyzed and were found in every infection within the study area. Frequencies for all 109 strains can be found in Table S1. Among the 5 common strains, strains 1 and 2 were the most closely related with only 4 mutational steps between them. Considering this in the context of relationships among strains, results indicate that strains 3, 4, and 5 are more distantly related to both strains 1 & 2 as well as each other.

### Genetic structure among infrapopulations

Pairwise comparisons among all parasite infrapopulations produced 4 clusters for both D_J-H_ and Φ_ST-H_ statistics (Figures S3 & S4, respectively). Both analyses clustered infrapopulations from the moderate transmission host villages into either a single cluster (D_J-H_) or two different clusters (Φ_ST-H_). The remaining two clusters contained infrapopulations from high transmission villages with the occasional exclusion of T0097PN from Peneng (only D_J-H_). T0557Y2 from Yautong2 also clustered with infrapopulations from moderate transmission villages in the case of Φ_ST-H_ statistic.

### Genetic structure among host villages

Infrapopulations within a host village were not significantly differentiated as given by AMOVA (Φ_ST-HV_; p = 0.134), pairwise Φ_ST-HV_, and pairwise D_J-HV_. Non-significant values suggest that infrapopulations within host villages may act as a single population. Following this assumption, infrapopulations occupying the same host villages were concatenated to facilitate a larger spatial scale analysis of genetic differentiation. In the unique case of Yautong2, where D_J-HV_ was significant, all analyses were conducted first with the infrapopulations separated and then concatenated.

**Figure 4:**
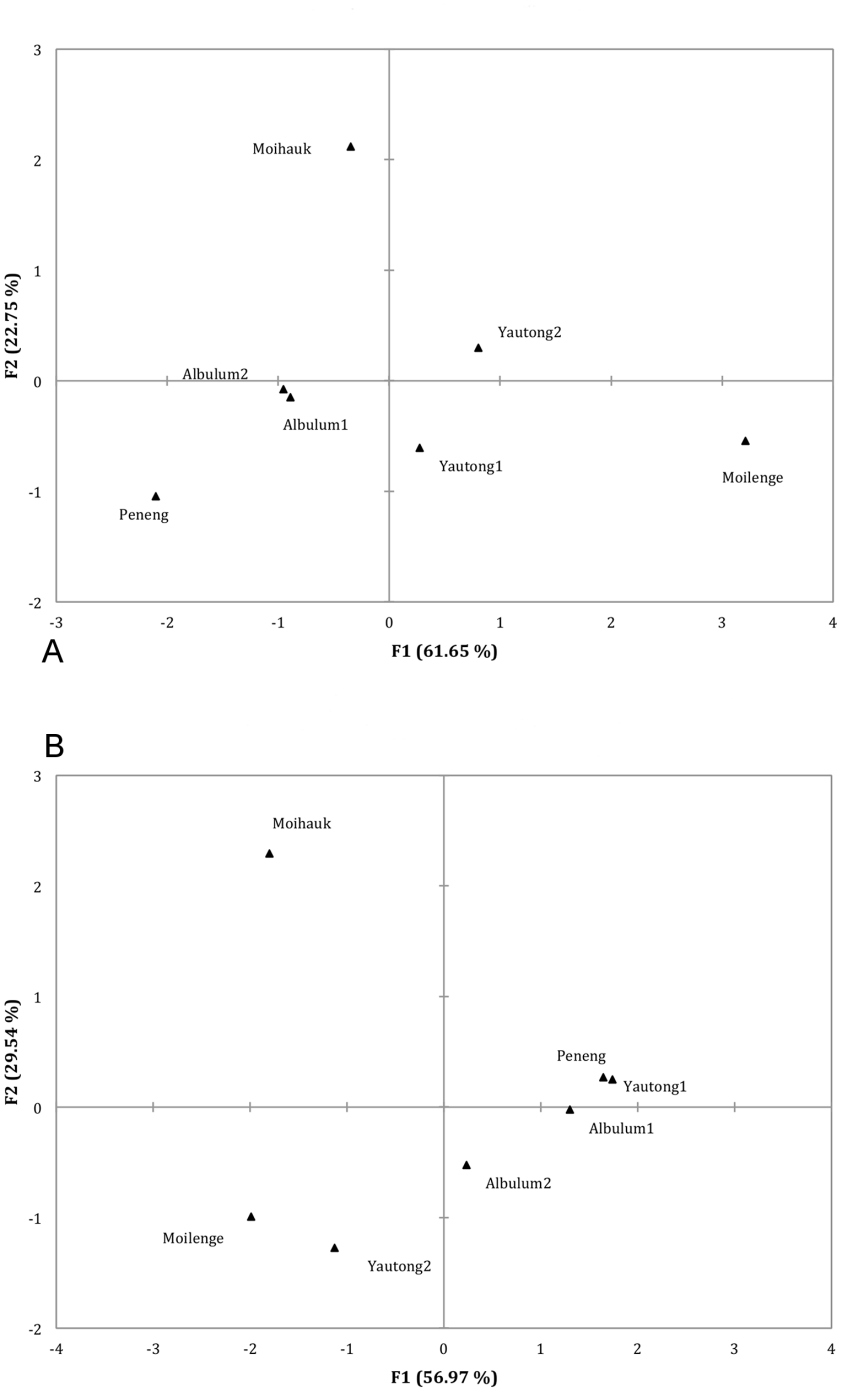
Multidimensional scaling analysis of genetic structure. Multidimensional scaling analysis of relationships among host villages based on genetic differentiation. We had the power to detect significant genetic differentiation between host villages 91% of the time when it existed [47]. Power for Φ_ST-V_ was <50% given a cut off of Φ_ST-V_ = 0. To have power equal to 90% we treated all values of Φ_ST-V_ relative to 0.0177 as undifferentiated. The X-axis and Y-axis represent the percent of variation explained by each dimension. **A**) Φ_ST-V_ the measure of genetic differentiation based on sequence distance [28] and **B**) D_J-V_ the measure of genetic differentiation based on allele frequencies [31].

Pairwise comparisons of both D_J-V_ and Φ_ST-V_ statistics among all host villages (Figure 4a & b, respectively) produced similar groupings where host villages that were geographically closer were more genetically similar. Consistently, host villages separated by greater distances were more genetically different. Represented as multidimensional scaling (MDS) plots, Figure 4 shows the spatial relations relative to values of genetic differentiation, D_J-V_ and Φ_ST-V_. For example Albulum1 and Albulum2 are only 0.5 kilometers distant and are genetically similar (D_J-V_ = 0.105; Φ_ST-V_ = 0.005), therefore on the MDS plot they are tightly clustered. Compare this to Peneng and Moilenge, which are geographically distant (∼9.5 kilometers), genetically different (D_J-V_ = 0.47; Φ_ST-V_ = 0.374) and are not clustered. Pairwise analysis using D_J-V_ was comparable to Φ_ST-V_, except in the placement of Yautong1 relative to Peneng.

### Reconstructing population history after an MDA

We found Tajima's D values non-significant for all infrapopulations (Table 1). When we examined Tajima's D at the host village level by grouping infrapopulations, we also did not find significant Tajima's D values. All Tajima's D values were also corroborated using a haplotype-based test of neutrality, Strobeck's *S* (data not shown) [37].

As we did not find values of Tajima's D concordant with a recent population contraction, we determined which parameter combinations would lead to a positive value of Tajima's D. If we assume that MDA reduces Wb effective population size by a given percentage each time the drugs are applied (once per year for 5 years), then we can calculate the amount of reduction needed per year to give a positive value of Tajima's D statistic. We constructed a simple model of Wb populations that assumed a single closed population with an effective population size of 300,000 individual Wb worms and 5 generations per year. We then subjected this population to 5 successive contractions, corresponding to the time period of the MDA in Wb generations (e.g. first treatment was 75 generations in the past), after which the population recovered to its current size. We found that only when we contracted the population by >90% each year for 5 consecutive years did we find a positive Tajima's D value.

## Discussion

We included 16 individuals from seven villages in the Dreikikir District, East Sepik Province of Papua New Guinea that all received five years of MDA [20]. The annual MDAs were effective at reducing MF prevalence in the study region [20]. However, a follow-up in 2008 found that MF prevalence had significantly increased from 1998 (Fisher's exact p<0.001) (Table 1), indicating a rebound in Wb populations.

To increase success of LF elimination we need to understand several parameters including *i)* the effectiveness of drug regimens, *ii)* the optimal time-course of drug administration, *iii)* the potential for drug resistance, *iv)* vector characteristics and *v*) human host migration dynamics [38]. At present, there are no experimental systems available to ask these questions directly in Wb, which is known to cause 90% of LF cases. However we can infer some of these parameters, indirectly, with genetic data. Through analyzing genetic data from parasite populations, there is potential for identifying, for example, strains that respond more slowly to drugs, or strains that demonstrate greater fecundity. Understanding strain-specific genetic differences could provide insight into how human population movement and mosquito species distributions influence Wb population structure. This information would enhance strategy development regarding the impact of MDA, such as how long to run an MDA program and the optimal size of the human population treatment unit.

### Genetic diversity

Our goal was to characterize the amount of genetic diversity in the overall parasite population of Wb in the in the Dreikikir District, East Sepik Province of PNG. As this is the first study of Wb population genetics in PNG we do not know the pre-MDA levels of genetic diversity and therefore we cannot objectively define if the diversity was affected by past treatments. Previous studies using DNA fingerprinting (RAPD) have shown that Wb populations were highly heterogeneous across India and Southeast Asia (H_T_ = 0.15; .20–.34) [39]–[43]. Also, Wb populations from Burkina Faso had similar heterozygosities when evaluated at a single SNP locus (H_e_ = 0.20,0.24,and 0.27) [15].

Within PNG, we may interpret the amount of genetic diversity by comparing it to a simplified expectation. First, we calculated an upper boundary on the expected amount of genetic diversity by assuming that Wb first colonized PNG 50,000–75,000 years ago and has a mutation rate equal to that of other nematodes (see [22]). Then assuming that there was a single isolated population that did not experience any population size changes, we estimate that genetic diversity should be at least equal to *θ* = 8.01 in the overall parasite population. The expected value was very close to what we observed for the overall parasite population in PNG *θ* = 7.83 (4.45–11.02).

At smaller spatial scales, we observed that diversity varied widely among infections with both high and low diversity found in the same village (Figure 2). We examined individual host variance by performing a multiple linear regression on the factors of age, location, and parasitemia. The number of sequences and parasitemia were both contributing factors and while we corrected for the number of sequences by subsampling (see Text S1 and Table S2 and S3), we could not correct for differences in parasitemia. While previous researchers have found positive correlations with parasitemia and the genetic diversity of an infection [16]; we found a strong but non-significant negative correlation.

The haplotype network provides additional perspective on the overall relationship between Wb strains in the study area. While it is interesting that strains 1 and 2 appear in every infection, in this study it was not possible to determine whether these strains are highly fecund, or may be resistant to drugs included in the MDA strategy. Despite this current limitation, we do know that the next MDA will have to reduce the prevalence of these strains if it hopes to be successful in eliminating Wb in the Dreikikir region. A haplotype network constructed during an ongoing MDA would essentially resemble a tree with few long branches (pruned), where rare strains are continually lost until only the most common strains (trunk) remain. The length of time needed for singleton strains to emerge in a treated population is not known. Therefore, it may be helpful to sample and genotype the Wb population at multiple time points throughout the course of MDA treatment and compare it to the pre-treatment population.

### Genetic structure

Wb parasites disperse across the landscape via movement of infected individuals, vector dispersal, or a combination of these factors. If infected individual people had recently moved to a new village we would expect their infrapopulation of parasites to be more closely related to their previous village. This pattern was not clearly evident in our data, as in most cases individuals from the same host village clustered together. There were exceptions in our dataset such as individuals T0145A2 from Albulum2 and T0557Y2 from Yautong2 that clustered more closely with infrapopulations from Moilenge (Figure 4S).

In other ecological systems the pattern of genetic diversity observed in Dreikikir is typically produced by short distance dispersal taking place over multiple generations [44]. Given the short flight of the Anopheline vectors in PNG (<2 km) and the distance between study villages (2 km apart), we cannot rule out the possibility that the pattern of genetic diversity was a function of vector dispersal. However we caution that the component of Wb dispersal attributable to human migration is uncertain, since high rates of human migration, in combination with high transmission, would quickly erase genetic differentiation between villages.

### Reconstructing population history after an MDA

The drugs currently used in MDA are effective against the parasite transmission stage (MF), but not as effective at killing adults [45]. If transmission is kept low over the entire lifetime of an adult worm, estimated to be 5 years [46], it would be theoretically possible to eliminate LF by delaying reproduction beyond the predicted life-time of adult worms. If large numbers of adult worms died during the last MDA in PNG then we would expect a population contraction. During a population contraction, genetic diversity is lost at a rate comparable to inverse of the effective population size, i.e. the smaller the population the faster diversity is lost. We chose Tajima's D statistic to evaluate whether the population had experienced a recent population contraction, which we would then interpret to be caused by the MDA. At the infrapopulation level the value of Tajima's D did not correspond with a recent or ongoing population contraction. Future studies utilizing more sequences, either from a second collection time-point or more independent genetic loci, would allow us to differentiate between competing scenarios.

### Summary

As genetic markers have not previously been available for Wb it follows that genetic diversity of the parasite has not been measured resulting in the inability to quantify Wb breeding population size or its potential for variation. With newly identified genetic markers it is now possible to use population genetics to assess potential for emergence of strains that are not responding to MDA strategies, and thereby monitor progress toward LF elimination by more than simple prevalence assessments. Here we have used population genetic data to infer that the parasite population is composed of many independent strains with overall high genetic diversity. We have also determined that the Wb population in Dreikikir is genetically structured. How vector dispersal, human migration and intervention have influenced this population structure remains to be determined. Beyond using these genetic markers to characterize basic population genetic characteristics of the Wb population in Dreikikier, we have shown how the frequency and distribution of polymorphisms can be used to evaluate the effects of a past MDA on genetic diversity. These results provide new capacity for evaluating and optimizing strategies for Wb elimination.

## Supporting Information

Figure S1 Relationship between the number of strains (haplotypes) and parasitemia (MF/ml).
(TIF)

Figure S2 Relationship between the number of strains (haplotypes) and the number of collected sequences from each individual samples.
(TIF)

Figure S3 Multidimensional Scaling plot of Jost's D statistic (D_J-H_) measuring genetic differentiation among parasite infrapopulations. An independent test using K-means algorithm in Genodive [48] produced highest support for 4 clusters; however using only a single locus limited the power to differentiate between the equally likely models of 3 clusters (AIC = −39.27) and 4 clusters (AIC = −39.25). Overall D_J-H_ supported a significant difference among all infrapopulations (D_J-H_ = 0.459, p<0.001).
(TIF)

Figure S4 Multidimensional Scaling plot of Φ_ST-H_ statistic measuring genetic differentiation among parasite infrapopulations. Overall Φ_ST_-_H_ supported a significant difference among all infrapopulations (Φ_ST-H_ = 0.172, p<0.001).
(TIF)

Table S1 Frequencies of all haplotypes observed in infrapopulation samples. Each infected individual is represented as a column heading with haplotype names as rows. Corresponding entries refer to the haplotype frequency found within each infrapopulation. Corresponding information on sample sizes and number of unique haplotypes can be found in Table 1.
(PDF)

Table S2 Cumulative density of the number of strains in each infrapopulation given a value of diversity, θ, and the current number of sequences. Projections to capture 95% of the total probability are also provided. Information on number of sequences and diversity are given in Table 1 and Figure 2.
(PDF)

Table S3 Cumulative density of the number of strains in each village level sample population given a value of diversity, θ, and number of sequences. Projections to capture 95% of the total probability are also provided.
(PDF)

Text S1 Supplemental text document containing additional details on methods used throughout the manuscript. Initial sections highlight data generation techniques such as sequencing and cloning while later sections detail data analysis. All methods parallel the main manuscript merely providing additional detail when needed.
(DOCX)
