# Supplementary figures and images for: Population Genetics of the Filarial Worm Wuchereria bancrofti in a Post-treatment Region of Papua New Guinea: Insights into Diversity and Life History
Source: PLoS Negl Trop Dis. 2013 Jul 11;7(7):e2308. doi: 10.1371/journal.pntd.0002308 (PMC3708868; doi:10.1371/journal.pntd.0002308)

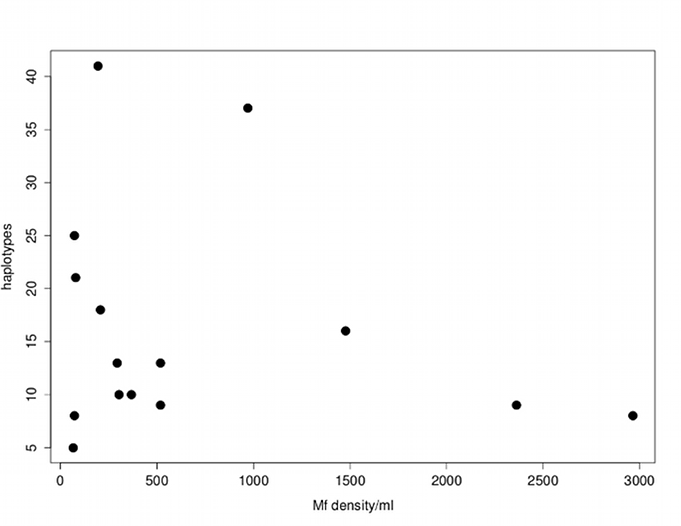

Supplement: Figure S1 — Relationship between the number of strains (haplotypes) and parasitemia (MF/ml). (TIF) [file pntd.0002308.s001.tif]

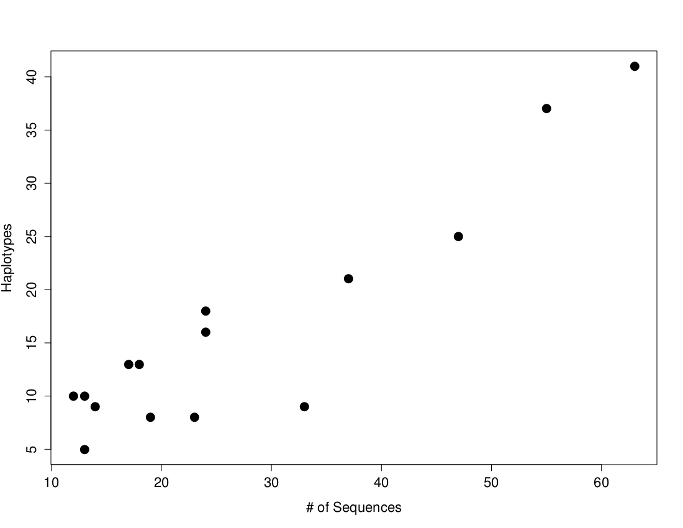

Supplement: Figure S2 — Relationship between the number of strains (haplotypes) and the number of collected sequences from each individual samples. (TIF) [file pntd.0002308.s002.tif]

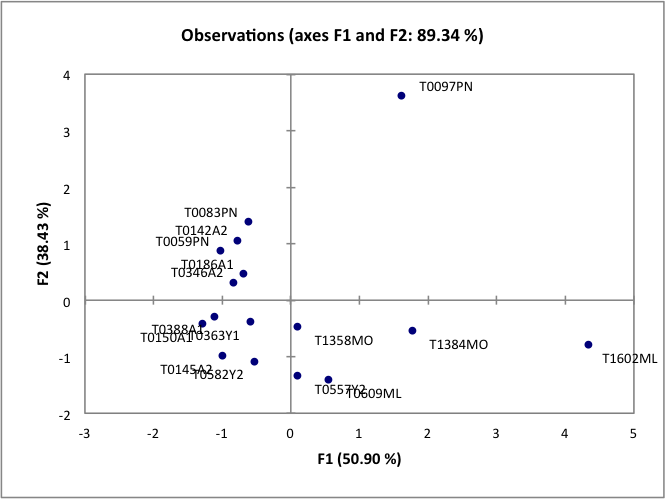

Supplement: Figure S3 — Multidimensional Scaling plot of Jost's D statistic (DJ-H) measuring genetic differentiation among parasite infrapopulations. An independent test using K-means algorithm in Genodive [48] produced highest support for 4 clusters; however using only a single locus limited the power to differentiate between the equally likely models of 3 clusters (AIC = −39.27) and 4 clusters (AIC = −39.25). Overall DJ-H supported a significant difference among all infrapopulations (DJ-H = 0.459, p<0.001). (TIF) [file pntd.0002308.s003.tif]

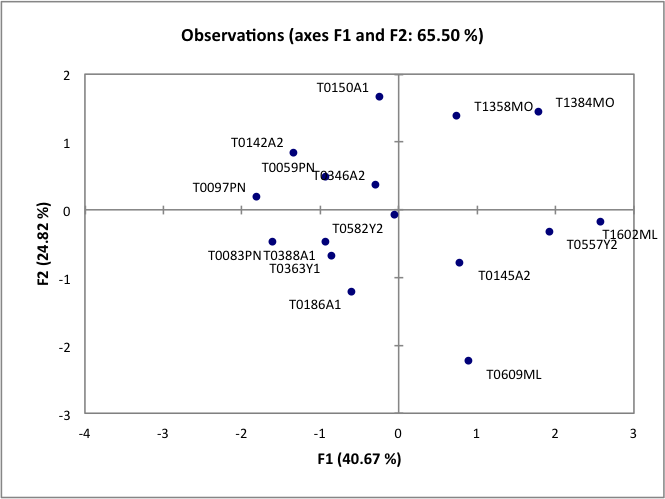

Supplement: Figure S4 — Multidimensional Scaling plot of ΦST-H statistic measuring genetic differentiation among parasite infrapopulations. Overall ΦST-H supported a significant difference among all infrapopulations (ΦST-H = 0.172, p<0.001). (TIF) [file pntd.0002308.s004.tif]
