# Supplementary material for: Population Genetics of the Filarial Worm Wuchereria bancrofti in a Post-treatment Region of Papua New Guinea: Insights into Diversity and Life History
Source: PLoS Negl Trop Dis. 2013 Jul 11;7(7):e2308. doi: 10.1371/journal.pntd.0002308 (PMC3708868; doi:10.1371/journal.pntd.0002308)
Supplement: Table S1 — Frequencies of all haplotypes observed in infrapopulation samples. Each infected individual is represented as a column heading with haplotype names as rows. Corresponding entries refer to the haplotype frequency found within each infrapopulation. Corresponding information on sample sizes and number of unique haplotypes can be found in Table 1. (PDF) [file pntd.0002308.s005.pdf]

**Table S1. Haplotype Frequencies**

Frequencies of all haplotypes observed in infrapopulation samples. Each infected individual is represented as a column heading with rows as haplotype names. Corresponding entries refer to the haplotype frequency found within each infrapopulation. Corresponding information on sample sizes and number of unique haplotypes can be found in Table 1.

| Name | Freq | T0059PN | T0083PN | T0097PN | T0150A1 | T0186A1 | T0388A1 | T0142A2 | T0145A2 | T0346A2 | T0363Y1 | T0557Y2 | T0582Y2 | T0609ML | T1602ML | T1358MO | T1384MO |
|---|---|---|---|---|---|---|---|---|---|---|---|---|---|---|---|---|---|
| Hap_2 (Strain 1) | 15 | 1 | 1 | 1 | 1 | 1 | 1 | 1 | 1 | 1 | 1 | | 1 | 1 | 1 | 1 | 1 |
| Hap_15 (Strain 2) | 13 | 1 | 1 | 1 | 1 | 1 | 1 | | 1 | 1 | 1 | 1 | 1 | 1 | | | 1 |
| Hap_13 (Strain 4) | 9 | 1 | | | 1 | | 1 | 1 | | 1 | | 1 | | 1 | | 1 | 1 |
| Hap_12 (Strain 3) | 7 | 1 | | | 1 | | 1 | | | 1 | | | 1 | | | 1 | 1 |
| Hap_3 (Strain 5) | 5 | | | | | | 1 | | | 1 | | | 1 | 1 | 1 | | |
| Hap_11 | 4 | | | | | | 1 | | | 1 | | 1 | | | | | 1 |
| Hap_9 | 3 | | | | 1 | | 1 | | | | | | | | 1 | | |
| Hap_43 | 3 | | | | | 1 | 1 | 1 | | | | | | | | | |
| Hap_32 | 3 | | | | 1 | | 1 | | | | | 1 | | | | | |
| Hap_26 | 3 | | | | | 1 | | | 1 | | | | 1 | | | | |
| Hap_24 | 3 | 1 | | | | | | 1 | | | | | 1 | | | | |
| Hap_22 | 3 | | | | | | | | 1 | | 1 | | | 1 | | | |
| Hap_8 | 2 | | | | | | 1 | | | | | | | | 1 | | |
| Hap_44 | 2 | | | | | | | | 1 | | 1 | | | | | | |
| Hap_40 | 2 | | | | 1 | | 1 | | | | | | | | | | |
| Hap_4 | 2 | | | | | | | | | | | | | 1 | 1 | | |
| Hap_25 | 2 | | | | 1 | | | | | | | | 1 | | | | |
| Hap_21 | 2 | | | | | | 1 | | | | | | | 1 | | | |
| Hap_19 | 2 | | | | | | 1 | | | | | | | | | 1 | |
| Hap_18 | 2 | | | | | | | | | 1 | | | | | | 1 | |
| Hap_17 | 2 | | 1 | | | | | | | | | | | | | | 1 |
| Hap_16 | 2 | 1 | | | | | | | | | | | | | | | 1 |
| Hap_99 | 1 | | | | | | | 1 | | | | | | | | | |
| Hap_98 | 1 | | | | | | | 1 | | | | | | | | | |
| Hap_97 | 1 | | | | | | | | 1 | | | | | | | | |
| Hap_96 | 1 | | | | | | | | 1 | | | | | | | | |
| Hap_95 | 1 | | | | | | | | 1 | | | | | | | | |
| Hap_94 | 1 | | | | | | | | 1 | | | | | | | | |
| Hap_93 | 1 | | | | 1 | | | | | | | | | | | | |
| Hap_92 | 1 | | | | 1 | | | | | | | | | | | | |
| Hap_91 | 1 | | | | 1 | | | | | | | | | | | | |
| Hap_90 | 1 | | | | 1 | | | | | | | | | | | | |
| Hap_89 | 1 | | | | 1 | | | | | | | | | | | | |
| Hap_88 | 1 | | | | 1 | | | | | | | | | | | | |
| Hap_87 | 1 | | | | 1 | | | | | | | | | | | | |
| Hap_86 | 1 | | | | 1 | | | | | | | | | | | | |
| Hap_85 | 1 | | | | 1 | | | | | | | | | | | | |
| Hap_84 | 1 | | | | 1 | | | | | | | | | | | | |
| Hap_83 | 1 | | | | 1 | | | | | | | | | | | | |
| Hap_82 | 1 | | | | 1 | | | | | | | | | | | | |
| Hap_81 | 1 | | | | 1 | | | | | | | | | | | | |
| Hap_80 | 1 | | | | 1 | | | | | | | | | | | | |
| Hap_79 | 1 | | | | 1 | | | | | | | | | | | | |
| Hap_78 | 1 | | | | 1 | | | | | | | | | | | | |
| Hap_77 | 1 | | | | 1 | | | | | | | | | | | | |
| Hap_76 | 1 | | | | 1 | | | | | | | | | | | | |
| Hap_75 | 1 | | | | 1 | | | | | | | | | | | | |
| Hap_74 | 1 | | | | 1 | | | | | | | | | | | | |
| Hap_73 | 1 | | | | 1 | | | | | | | | | | | | |
| Hap_72 | 1 | | | | | 1 | | | | | | | | | | | |
| Hap_71 | 1 | | | | | 1 | | | | | | | | | | | |
| Hap_70 | 1 | | | | | | | | | 1 | | | | | | | |
| Hap_7 | 1 | | | | | | | | | | | | | | 1 | | |
| Hap_69 | 1 | | | | | | | | | 1 | | | | | | | |
| Hap_68 | 1 | | | | | | | | | 1 | | | | | | | |
| Hap_67 | 1 | | | | | | | | | 1 | | | | | | | |
| Hap_66 | 1 | | | | | | | | | 1 | | | | | | | |
| Hap_65 | 1 | | | | | | | | | 1 | | | | | | | |
| Hap_64 | 1 | | | | | | | | | 1 | | | | | | | |
| Hap_63 | 1 | | | | | | | | | 1 | | | | | | | |
| Hap_62 | 1 | | | | | | | | | 1 | | | | | | | |
| Hap_61 | 1 | | | | | | | | | 1 | | | | | | | |
| Hap_60 | 1 | | | | | | | | | 1 | | | | | | | |
| Hap_6 | 1 | | | | | | | | | | | | | | 1 | | |
| Hap_59 | 1 | | | | | | | | | 1 | | | | | | | |
| Hap_58 | 1 | | | | | | | | | 1 | | | | | | | |
| Hap_57 | 1 | | | | | | | | | 1 | | | | | | | |
| Hap_56 | 1 | | | | | | | | | 1 | | | | | | | |
| Hap_55 | 1 | | | | | | | | | 1 | | | | | | | |
| Hap_54 | 1 | | | | | | | | | 1 | | | | | | | |
| Hap_53 | 1 | | | | | | | | | 1 | | | | | | | |
| Hap_52 | 1 | | | | | | | | | 1 | | | | | | | |
| Hap_51 | 1 | | | | | | | | | 1 | | | | | | | |
| Hap_50 | 1 | | | | | | | | | 1 | | | | | | | |
| Hap_5 | 1 | | | | | | | | | | | | | | 1 | | |
| Hap_49 | 1 | | | | | | | | | 1 | | | | | | | |
| Hap_48 | 1 | | | | | | | | | | 1 | | | | | | |
| Hap_47 | 1 | | | | | | | | | | 1 | | | | | | |
| Hap_46 | 1 | | | | | | | | | | 1 | | | | | | |
| Hap_45 | 1 | | | | | | | | | | 1 | | | | | | |
| Hap_42 | 1 | | | | | | 1 | | | | | | | | | | |
| Hap_41 | 1 | | | | | | 1 | | | | | | | | | | |
| Hap_39 | 1 | | | | | | 1 | | | | | | | | | | |
| Hap_38 | 1 | | | | | | 1 | | | | | | | | | | |
| Hap_37 | 1 | | | | | | 1 | | | | | | | | | | |

| | | | | | | | | | | | | | | | | | |
|---|---|---|---|---|---|---|---|---|---|---|---|---|---|---|---|---|---|
| Hap_36 | 1 | | | | | | 1 | | | | | | | | | | |
| Hap_35 | 1 | | | | | | | | | | | 1 | | | | | |
| Hap_34 | 1 | | | | | | | | | | | 1 | | | | | |
| Hap_33 | 1 | | | | | | | | | | | 1 | | | | | |
| Hap_31 | 1 | | | | | | | | | | | 1 | | | | | |
| Hap_30 | 1 | | | | | | | | | | | | 1 | | | | |
| Hap_29 | 1 | | | | | | | | | | | | 1 | | | | |
| Hap_28 | 1 | | | | | | | | | | | | 1 | | | | |
| Hap_27 | 1 | | | | | | | | | | | | 1 | | | | |
| Hap_23 | 1 | | | | | | | | | | | | 1 | | | | |
| Hap_20 | 1 | | | | | | | | | | | | | 1 | | | |
| Hap_14 | 1 | | | | | | | | | | | | | | | | 1 |
| Hap_109 | 1 | 1 | | | | | | | | | | | | | | | |
| Hap_108 | 1 | 1 | | | | | | | | | | | | | | | |
| Hap_107 | 1 | 1 | | | | | | | | | | | | | | | |
| Hap_106 | 1 | | 1 | | | | | | | | | | | | | | |
| Hap_105 | 1 | | | | | | | 1 | | | | | | | | | |
| Hap_104 | 1 | | | | | | | 1 | | | | | | | | | |
| Hap_103 | 1 | | | | | | | 1 | | | | | | | | | |
| Hap_102 | 1 | | | | | | | 1 | | | | | | | | | |
| Hap_101 | 1 | | | | | | | 1 | | | | | | | | | |
| Hap_100 | 1 | | | | | | | 1 | | | | | | | | | |
| Hap_10 | 1 | | | | | | | | | | | | | | 1 | | |
| Hap_1 | 1 | | | | | | | | | | | | | | 1 | | |
