# Supplementary material for: Population Genetics of the Filarial Worm Wuchereria bancrofti in a Post-treatment Region of Papua New Guinea: Insights into Diversity and Life History
Source: PLoS Negl Trop Dis. 2013 Jul 11;7(7):e2308. doi: 10.1371/journal.pntd.0002308 (PMC3708868; doi:10.1371/journal.pntd.0002308)
Supplement: Table S2 — Cumulative density of the number of strains in each infrapopulation given a value of diversity, θ, and the current number of sequences. Projections to capture 95% of the total probability are also provided. Information on number of sequences and diversity are given in Table 1 and Figure 2. (PDF) [file pntd.0002308.s006.pdf]

Table S2. Sequence sampling to assess haplotype diversity of infrapopulations

Cumulative density of each infrapopulation given a value of diversity, θ, and the current number of sequences. Projections to capture 95% of the total probablity are also provided. Information on number of sequences and diversity are given in Table 1 and Figure 2.

	T0059PN	T0083PN	T0097PN	T0346A2	T0142A2	T0145A2	T0150A1	T0186A1	T0388A1	T0363Y1	T0557Y2	T0582Y2	T0609ML	T1602ML	T1358MO	T1384MO
ϑ	5.15	4.91	1	6.88	3.94	6.87	4.30	5.89	7.64	5.40	7.33	7.29	6.94	5.66	9.12	5.01
Number of sequences/individual	37	19	13	55	17	75	47	24	36	23	18	24	12	13	33	14
Cumulative Density	0.87	0.77	0.92	0.88	0.79	0.91	0.91	0.78	0.81	0.79	0.66	0.73	0.55	0.64	0.76	0.69
Number of sequences/individual to 95% capture	100	96	20	135	77	134	84	115	149	106	143	143	136	111	178	98
