# Supplementary material for: Population Genetics of the Filarial Worm Wuchereria bancrofti in a Post-treatment Region of Papua New Guinea: Insights into Diversity and Life History
Source: PLoS Negl Trop Dis. 2013 Jul 11;7(7):e2308. doi: 10.1371/journal.pntd.0002308 (PMC3708868; doi:10.1371/journal.pntd.0002308)
Supplement: Table S3 — Cumulative density of the number of strains in each village level sample population given a value of diversity, θ, and number of sequences. Projections to capture 95% of the total probability are also provided. (PDF) [file pntd.0002308.s007.pdf]

Table S3. Sequence sampling to assess haplotype (strain) diversity of Villages

Cumulative density of each village level sample population given a value of diversity, θ, and number of sequences. Projections to capture 95% of the total probablity are also provided.

Village	Peneng	Albulum2	Albulum1	Yautong1	Yautong2	Moilenge	Moihauk
ϑ	4.31	6.58	7.14	5.40	6.48	5.23	6.44
Number of sequences/village	69	134	147	23	42	25	47
Cumulative Density	0.94	0.95	0.95	0.79	0.86	0.81	0.87
Number of sequences/village to 95%	84	129	140	106	127	103	126
