# Supplementary material for: Population Genetics of the Filarial Worm Wuchereria bancrofti in a Post-treatment Region of Papua New Guinea: Insights into Diversity and Life History
Source: PLoS Negl Trop Dis. 2013 Jul 11;7(7):e2308. doi: 10.1371/journal.pntd.0002308 (PMC3708868; doi:10.1371/journal.pntd.0002308)
Supplement: Text S1 — Supplemental text document containing additional details on methods used throughout the manuscript. Initial sections highlight data generation techniques such as sequencing and cloning while later sections detail data analysis. All methods parallel the main manuscript merely providing additional detail when needed. (DOCX) [file pntd.0002308.s008.docx]

**Population Genetics of the filarial worm *Wuchereria bancrofti* in a Post-treatment region of Papua New Guinea: Insights into diversity and life history**

Scott T. Small, Akshaya Ramesh, Krufinta Bun, Lisa Reimer, Edward Thomsen, Manasseh Baea, Moses Bockarie, Peter Siba, James W. Kazura, Daniel J. Tisch, Peter A. Zimmerman.

**Supporting Information**

**Genomic DNA extraction and PCR amplification**

Genomic DNA (gDNA) was extracted from whole blood using a QIAamp 96 DNA Blood Kit (QIAGEN, Valencia, CA). PCR Primers were designed to amplify 650 bp of the partial *cox1* [1]. PCR master mixtures (25 μL) contained 2.5 μL 10X buffer (16.6 mM (NH_4_)_2_SO_4_, 10 mM β-mercaptoethanol, 3.4 or 6.7 mM MgSO_4_, and 67 mM Tris-HCl, pH 8.8), 2 μL dNTPs (5 nmol each dNTP), 0.6 μL each primer (6 pmol), 0.6 μL Taq DNA polymerase (2.5 units) and 1 μL of DNA template. An MJ Research PTC-225 thermocycler (MJ Research, Waltham, MA) was used to amplify the *cox1* fragments under the thermocycling conditions: 92°C for 2 min (initial denaturation), followed by 40 cycles of denaturation at 92°C for 30s, annealing at 50°C for 30s, and extension at 72°C for 60 s followed by a final extension at 72°C for 5 minutes. The amplification products were visualized on 2% (w/v) agarose gels and the gels were stained with SYBR Gold (Molecular Probes, Eugene, OR) and visualized using a Storm 860 molecular imaging system with ImageQuant 5.2 software (Molecular Dynamics, Sunnyvale, CA). These PCR products were TA cloned into pCR2.1-TOPO vector and transformed into chemically competent *E. coli* TOP10 cells according to the manufacturer’s protocol (Invitrogen, Carlsbad, CA).

**Sanger Sequencing**

All viable clones were selected and size screened via gel electrophoresis for the PCR insert. Clones containing the insert were then cleaned with an enzyme reaction, exo-sap. The exo-sap master mixture (10 μL) contained 0.1 μL exonuclease, 0.5 μL antarctic phosphatase and 7 μL of PCR product. The reaction was then run in a MJ Research PTC-225 thermocycler (MJ Research, Waltham, MA) under the thermo-cycling conditions: 37°C for 30 min followed by 95°C for 5 min. Samples were then diluted to 2ng/ µl per 100 bp concentration and prepared for cycle sequencing. Cycle sequencing used a modified reaction protocol with 2µl of 5X Sequencing buffer, 1µl of Big dye terminator, 0.36µl of primer (3.2 pmol), 2.4µl of water and 4 µl of diluted PCR product from the exo-sap reaction. The thermo-cycling condition for cycle sequencing was an initial denaturation at 96°C for 30s followed by 25 cycles of denaturation at 96°C for 10 seconds, annealing at 50°C for 5 seconds and the extension step at 60°C for 4 min.

Following cycle-sequencing reaction, PCR products were purified to remove unincorporated dyes and single stranded DNA. Purification was performed by adding 2.5μl of 125mM EDTA followed by 30μL of 100% ethanol to each sample. Samples were then mixed by inversion, incubated at room temperature for 15 minutes, and centrifuged at 3000g for 30 min at 4 degrees C. Next, the supernatant was discarded and 30μL of 70% ethanol was added to the individual samples and centrifuged for 15 minutes at 1650g. The supernatant was then discarded and placed in a vacuum chamber for 15 minutes to ensure complete drying. Once dry, 10μl of HiDi formamide (Applied Biosystems, Foster City, CA) was added to individual wells and loaded onto the ABI PRISM 3700 DNA Sequencer (Applied Biosystems, Foster City, CA).

**Sequence processing**

All sequences were edited and assembled using CodonCode Aligner 3.5 (CodonCode Corporation, Dedham MA). The ends of the sequences were clipped and remaining sites, with PHRED scores <30, were visually investigated and recorded as ambiguities if not clearly classified. Sequences with less than a minimum of 300 high quality bases (PHRED score >30) were removed from the assembly. Edited sequences were then imported into Geneious 5.3 and assembled against the complete mitochondrial genome of *W. bancrofti* (GenBank Accession No. JF 557722) to verify that all the sequences were in identical translation frames and no stop codons were introduced. After verifying that sequences were in translation frame, a correction for Taq DNA polymerase errors was applied to sequences within each individual sample sequence assembly. Using an infinite series model [2] and assuming a Poisson distribution, the expected number of pairwise differences (*π*) between any two sequences can be approximated by

$$\pi\approx2n\mu G \frac{\beta}{1+\beta}$$

where *n* is the number of PCR cycles, *µ* is the *Taq* polymerase error rate, *G* is the length of the sequence, and *ß* is the efficiency of *Taq* polymerase [3]. Using the above equation, the expected number of pairwise differences due to *Taq* error alone was 1.67. Therefore, polymorphisms that occurred less than twice in the infrapopulation sequence assemblies were treated as *Taq* DNA polymerase errors and modified to match the consensus sequence. An independent validation using a single L3 stage larvae with 30 sampled sequences, verified that 86% of sequences had 0 errors while 14% had at least 1 error. Post-correction, our L3 sequences yielded a single haplotype*.*

After correcting polymerase errors, UCHIME, a chimera detection program, was used to detect PCR based recombinant molecules [4]. The UCHIME algorithm divides the input sequences into four non-overlapping sequences (chunks) and builds a reference database based on the consensus sequence. The algorithm then matches these chunks to the reference database and selects two of the best matches to generate a three-way multiple alignment. Based on the percent identity of the alignment, a score is assigned and a chimeric molecule is reported if the score exceeds a pre-determined threshold [4]. Sequences reported as chimeric were subsequently removed before further analysis.

**Multiple linear regression and predicting number of strains**
The number of Wb strains in a host was best defined by parasitemia and the number of sequences collected (AIC= 55.14, R^2^=0.775, p<0.001). A test for outlying data points given by Cook’s distance [5] revealed that removing sample T0388A1 would improve the fit of the regression; removal of T0388A1 improved the fit of the model (AIC=37.74, R­^2^=0.91, p-<0.001). The new data set included host village and transmission rate as predictive factors. However a test for multicollinearity revealed that these were highly correlated, so only host village was included. Including the age of host in the model did not improve the overall fit to the new data set.

An analysis of variance (ANOVA) was performed to measure the effect of each variable on the number of strains. Only parasitemia (p-value=0.038) and the number of sequences (p <0.001) were found to contribute to the variance. Host village was not a factor (p = 0.219), however a fixed effects model attributed a small amount of variance to the host village of Peneng (p < 0.05). The number of strains was negatively correlated with parasitemia (as MF density) (Pearson correlation r= -0.22, p=0.04132), while the number of strains was positively correlated with the number of sequences (Pearson correlation r=0.8786, p<0.001) (Figures S1 & S2). The data did not violate any of the assumptions of linear models.

**Population saturation and sampling**

A single sample, T0388, was chosen to be isolated/cloned/sequenced 3 independent times. Our analysis of the 3 independent samples was consistent for haplotypes with frequencies >0.1. Rare haplotypes (<0.1) were recovered with varying success and found to be highly dependent on the number of clones captured and sequenced. To determine the percent of rare haplotypes captured,

we used a subsample of our sequences to estimate the population mutation rate θ. Under a given value of θ, the distribution of haplotypes/alleles in a population is best described by Ewen’s sampling formula (ESF) which gives the configuration of the number of times each allele is observed [6]. Using the density function of ESF, we calculate the cumulative probability (% of total haplotypes sampled) for our current samples sizes as well as the sample size needed to “capture” 95% of the expected haplotypes. We, on average, capture 78% of the total expected haplotypes for the 16 infrapopulations and 88% of the total number of expected haplotypes for the 7 villages. Supplemental tables S1 and S2 detail the cumulative probability associated with sampling each infrapopulation and village, respectively.

**Sequence subsampling for statistical analysis**

To account for inequality in sample sizes among infrapopulations, an artifact of sequence collection and cloning efficiency, we randomly subsampled all infrapopulations before calculating genetic statistics. Infrapopulations were randomly sampled by drawing 10 sequences with replacement. The new subsampled populations were then used to calculate all genetic statistics. This process was repeated 1000 times with the resulting distribution used to determine the mean and variance of each statistic.

**Grouping infrapopulations within a host village**

Interpretation of Φ_ST-V_ was dependent on comparing the null distribution created by random sampling from a pool of all infrapopulations. Φ_ST-V_ values for panmictic simulations overlapped in all host villages with estimated Φ_ST-V ­_values among real infrapopulations. Interpretation of D_J-HV_ was dependent on a 1000 step permutation test where H_0_ was that D_J-V_=0—panmixia. Within host villages, the permutation test failed to reject panmixia (Bonferroni adjusted α=0.0083 for Peneng, Albulum1, and Albulum2) except in case of Yautong2 (p-value =0.0195). Differences between infrapopulations within Yautong 2 were also reflected in the infrapopulation clustering analysis that consistently placed T0557Y2 and T0582Y2 into separate groups; no significant difference was found using Φ_ST-V_.

**Power tests of genetic differentiation**

The program POWSIM [7], which calculates power for the correlate statistic of F_ST_ [8], was used to assess power to detect genetic differentiation and therefore reject panmixia (POWSIM parameters: 10 generations per year, N_e_=5000-10000, 10 years of drift with 10 generations per year—power was lowered (<80%) when we assumed fewer than 10 generations per year). Power analysis was also determined directly for Φ_ST-V_ by generating a null distribution of Φ_ST_ for each host village, recording the proportion of simulations where Φ_ST-V_ was great than 0, and then subtracting this value from 1.

**References**

1. Ramesh A, Small ST, Kloos ZA, Kazura JW, Nutman TB, et al. (2012) The complete mitochondrial genome sequence of the filarial nematode Wuchereria bancrofti from three geographic isolates provides evidence of complex demographic history. Molecular Biochemical Parasitology 183: 32–41.

2. Watterson G (1975) On the number of segregating sites in genetical models without recombination. Theoretical Population Biology 7: 265–276.

3. Krawczak M, Reiss J (1989) Polymerase chain reaction: replication errors and reliability of gene diagnosis. Nucleic acids … 17: 2197–2201.

4. Edgar R, Haas B, Clemente J (2011) UCHIME improves sensitivity and speed of chimera detection. … 27: 2194–2200. doi:10.1093/bioinformatics/btr381.

5. Cook RD, Sanford W (1982) Residuals and influence in regression. New York, NY: Chapman & Hall.

6. Ewens W (1972) The sampling theory of selectively neutral alleles. Theoretical population biology 3: 87–112.

7. Ryman N, Palm S (2006) POWSIM: a computer program for assessing statistical power when testing for genetic differentiation. Molecular Ecology Notes 6: 600–602. doi:10.1111/j.1365-294X.2006.01378.x.

8. Wright S, MePhee HC (1925) An approximate method of calculating coefficients of inbreeding and relationship. J Agric Res 31: 377–383.
